# Contrast-Related Acute Pulmonary Edema Following Diagnostic Coronary Angiography: A Case Report

**DOI:** 10.7759/cureus.102699

**Published:** 2026-01-31

**Authors:** Ayobami B Omodara, Naveeda Mahar, Annabelle Milorde Attolico, Ayomide D Sanyaolu

**Affiliations:** 1 Cardiology, Mid and South Essex NHS Foundation Trust, Southend-on-Sea, GBR; 2 Acute Medicine, Mid and South Essex NHS Foundation Trust, Southend-on-Sea, GBR; 3 Internal Medicine, Southend University Hospital, Southend-on-Sea, GBR; 4 Respiratory Medicine, Southend University Hospital, Southend-on-Sea, GBR

**Keywords:** acute pulmonary edema, adverse side effect, cardiogenic pulmonary edema, contrast-induced acute lung injury, contrast-induced complications, contrast-related adverse events, ncpe, noncardiogenic pulmonary edema

## Abstract

Acute pulmonary edema (APE) secondary to diagnostic angiography is an uncommon complication of the procedure. It may result from several factors, including underlying congestive cardiac failure with poor left ventricular (LV) systolic function, baseline fluid overload, excessive use of contrast during diagnostic angiography in patients with severe coronary artery disease and LV impairment, and, very rarely, an anaphylactoid reaction to iodine-containing intravenous contrast agents. We report the case of a 60-year-old man who presented to the ED with an intermittent history of worsening nonexertional chest pain associated with breathlessness, occasionally relieved by rest and/or glyceryl trinitrate (GTN) spray. The chest pain was also worse following heavy meals. Due to the infrequent nature of his symptoms, he was seen in a cardiology clinic and referred for an urgent outpatient CT coronary angiogram, which suggested three-vessel disease, primarily involving the right coronary artery and left anterior descending artery, with milder disease in the left circumflex artery. Echocardiography revealed mildly reduced LV systolic function (LV ejection fraction = 45%). In the weeks leading up to his invasive angiogram, he noted mild ankle swelling and orthopnea, for which his general practitioner prescribed oral furosemide 40 mg daily for two weeks. He appeared euvolemic on the day of the angiogram. The coronary angiogram was uneventful, confirming severe multivessel coronary disease. Within 10 minutes of the procedure, the patient developed APE, suspected based on subtle angiographic findings, physical examination, and chest X-ray. He was promptly started on intravenous GTN infusion and made a good clinical recovery within 48 hours. He was later transferred to a tertiary center for coronary artery bypass graft surgery.

## Introduction

Acute pulmonary edema (APE) is a life-threatening condition characterized by the rapid accumulation of fluid in the pulmonary interstitium and alveoli, leading to impaired gas exchange and severe respiratory compromise. While commonly associated with acute myocardial infarction, severe valvular heart disease, or exacerbations of chronic heart failure, APE as a complication of diagnostic coronary angiography is uncommon and relatively underreported in the literature [1]. Diagnostic coronary angiography is an invasive imaging procedure that uses contrast dye and X-ray fluoroscopy to visualize the coronary arteries, allowing clinicians to assess for blockages, narrowing, or other structural abnormalities of the heart’s blood vessels.

Coronary angiography, which can be invasive or noninvasive, is a cornerstone in the diagnosis and management of ischemic heart disease, providing detailed visualization of coronary anatomy and guiding revascularization strategies [2]. Although generally considered safe, an invasive coronary angiogram carries procedural risks, including contrast-induced nephropathy, arrhythmias, vascular complications, and, rarely, acute cardiac decompensation. In certain patients, particularly those with preexisting left ventricular (LV) dysfunction, fluid overload, or extensive coronary artery disease (CAD), the administration of contrast media can precipitate APE.

The pathophysiology is multifactorial and may involve transient myocardial ischemia, contrast-induced fluid shifts, and increased LV filling pressures, as measured by LV end-diastolic pressure (LVEDP), ultimately overwhelming cardiac compensatory mechanisms [3]. With older contrast agents, the negative inotropic effects of hyperosmolar media caused by fluid redistribution have been documented, leading to a similar rise in LVEDP. As suspected in our case, multivessel CAD, a mildly to moderately elevated LVEDP of 26 mmHg, and an LV systolic function of approximately 45% were predisposing factors that contributed to the development of APE.

The rarity of APE following diagnostic coronary angiography may be partly attributable to careful procedural planning, including the identification of high-risk individuals (e.g., those with poor LV systolic function), as well as the widespread adoption of low-osmolar, nonionic iodinated contrast agents, which are generally better tolerated. Despite being a recognized but exceptionally rare complication of diagnostic coronary angiography, contrast-related APE is poorly represented in the published literature. Existing knowledge is largely confined to isolated case reports, with no systematic studies or clear management guidance available. This scarcity of evidence highlights a significant gap in understanding and underscores the value of individual case reports in informing clinical awareness and practice.

## Case presentation

A 60-year-old man initially presented to the ED with intermittent atypical chest pain. The pain was variable in character and occurred both during exertion and after meals, particularly when the patient lay down soon afterward. Episodes also recurred during periods of occupational stress, despite his work being physically undemanding. The pain was typically brief, although on rare occasions it persisted for most of the day.

Important differential diagnoses were considered and excluded. There was no fever or cough to suggest a chest infection, inflammatory markers were normal, and his Wells score for deep vein thrombosis and pulmonary embolism (PE) was 2 (very low), with a negative D-dimer at the time. He reported no recent viral respiratory infection and no pleuritic or pericarditic chest pain. Physical examination revealed normal pleural sounds and no pericardial rub. Vital signs, including blood pressure, were within normal limits. Initial investigations showed unremarkable dynamic troponin levels, no ischemic changes on ECG, and an unremarkable chest X-ray (CXR).

The patient was a lifelong nonsmoker, did not consume alcohol, and denied any use of illicit substances. He was independent in daily activities and had no prior history of CAD, atherosclerotic disease, hypertension, diabetes mellitus, or regular medication use. He was administered omeprazole, sublingual glyceryl trinitrate (GTN) spray, and paracetamol, which helped relieve his symptoms. Due to the atypical nature of his presentation, he was discharged from the ED with a suspected diagnosis of gastroesophageal reflux disease, with a plan to rule out an atypical presentation of significant CAD. He was referred to the cardiology outpatient chest pain clinic for further assessment.

A few weeks later, he was reviewed in the cardiology clinic. Based on an intermediate pretest probability score for CAD, a CT coronary angiogram (CTCA) was arranged. The CTCA demonstrated three-vessel coronary disease, with significant stenosis involving the proximal right coronary artery (RCA) and left anterior descending (LAD) artery. In view of these findings and persistent symptoms, an urgent invasive diagnostic coronary angiography was planned. An echocardiogram was arranged prior to the invasive procedure.

The echocardiogram showed mild LV dilation (LVIDd = 5.8 cm (normal <5.6 cm); LVIDs = 4.1 cm), with mild hypokinesia of the inferior and posterior walls. LV ejection fraction (LVEF) was visually estimated at 45%. Right ventricular function was normal, with no significant valvular disease or regurgitation. There was no evidence of pericardial or aortic root pathology. The inferior vena cava was collapsible >50%.

On admission for his elective outpatient diagnostic coronary angiogram, a focused physical and cardiovascular examination revealed a regular pulse of 72 beats per minute, normal heart sounds (S1 and S2) without added sounds or murmurs, and no jugular venous distention. There was no significant pitting edema. Blood pressure was 124/68 mmHg on the left arm and 128/70 mmHg on the right arm. Respiratory examination showed a respiratory rate of 17 breaths per minute, vesicular breath sounds in all lung fields, and oxygen saturation of 98%. The temperature was 36.7°C. Blood tests from his recent ED visit were unremarkable, with normal renal function (estimated glomerular filtration rate >90 mL/min) and electrolytes within normal limits.

He reported a history of mild ankle swelling and orthopnea, for which his general practitioner prescribed oral furosemide 40 mg daily for two weeks prior to cardiology clinic attendance. He denied paroxysmal nocturnal dyspnea or palpitations and appeared euvolemic.

**Figure 1:**
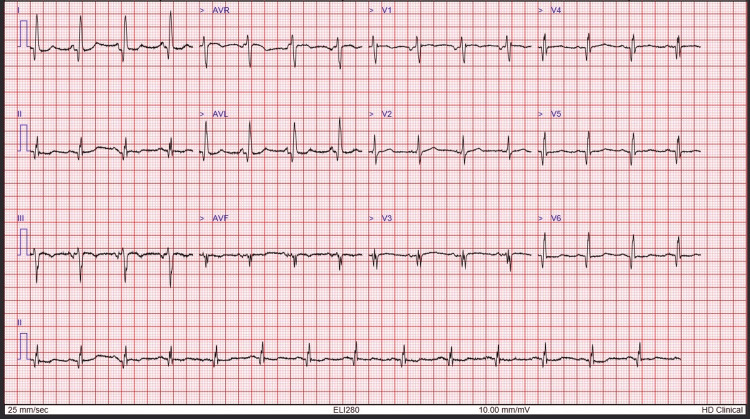
ECG ECG demonstrating sinus rhythm with prominent Q waves in anterolateral leads (V3-V6), high lateral leads (I, aVL), and inferior leads (II, aVF), suggesting completed infarction in the corresponding myocardial segments.

A routine ECG was performed (Figure 1).

**Figure 2:**
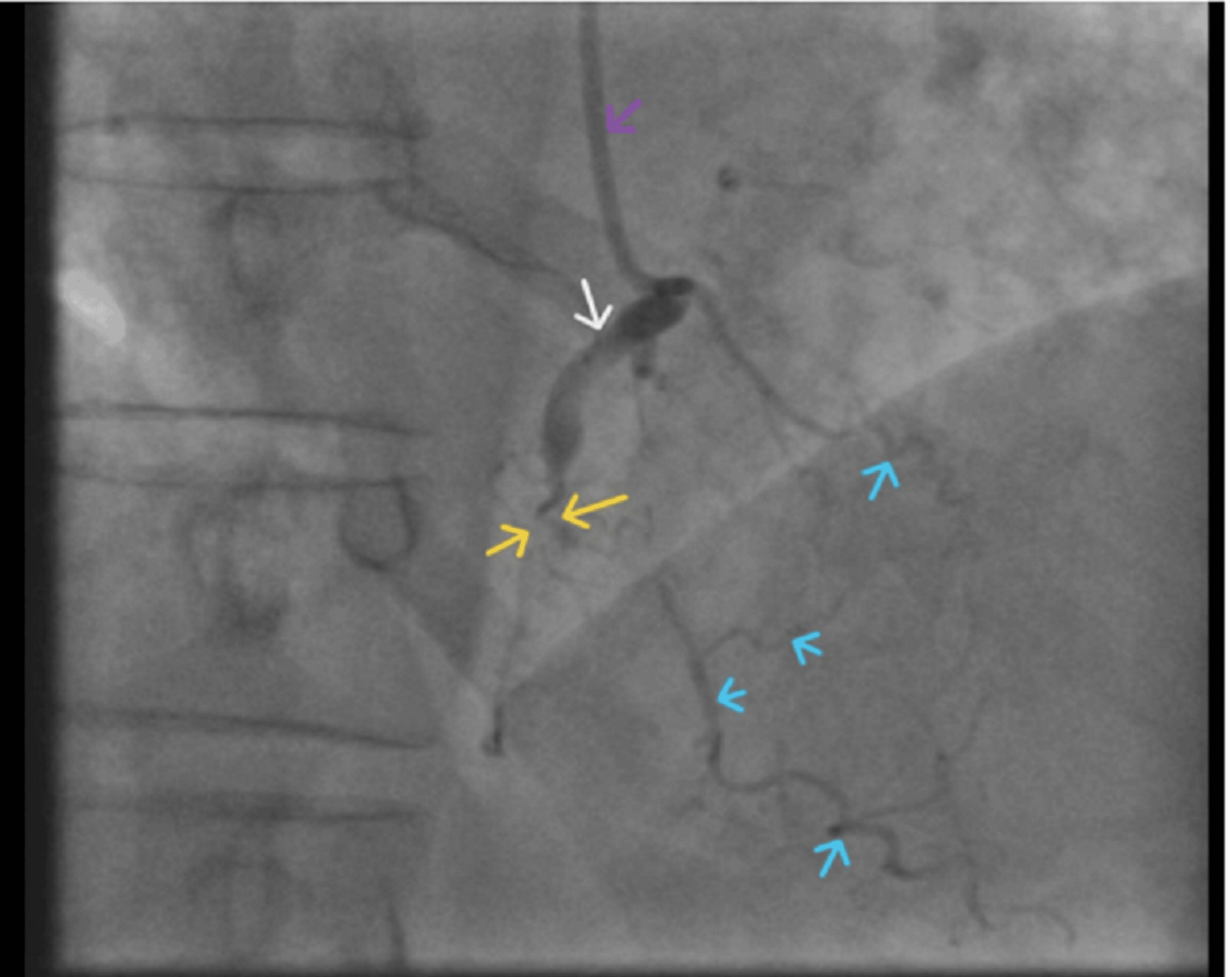
Coronary angiogram Coronary angiogram showing near-total occlusion of the RCA (yellow arrow). Blue arrows indicate backfilling from the LAD artery and collateral vessels. The proximal segment of the RCA is marked by the white arrow. The purple arrow denotes the Judkins Right 4.0 catheter. LAD, left anterior descending; RCA, right coronary artery

**Figure 3:**
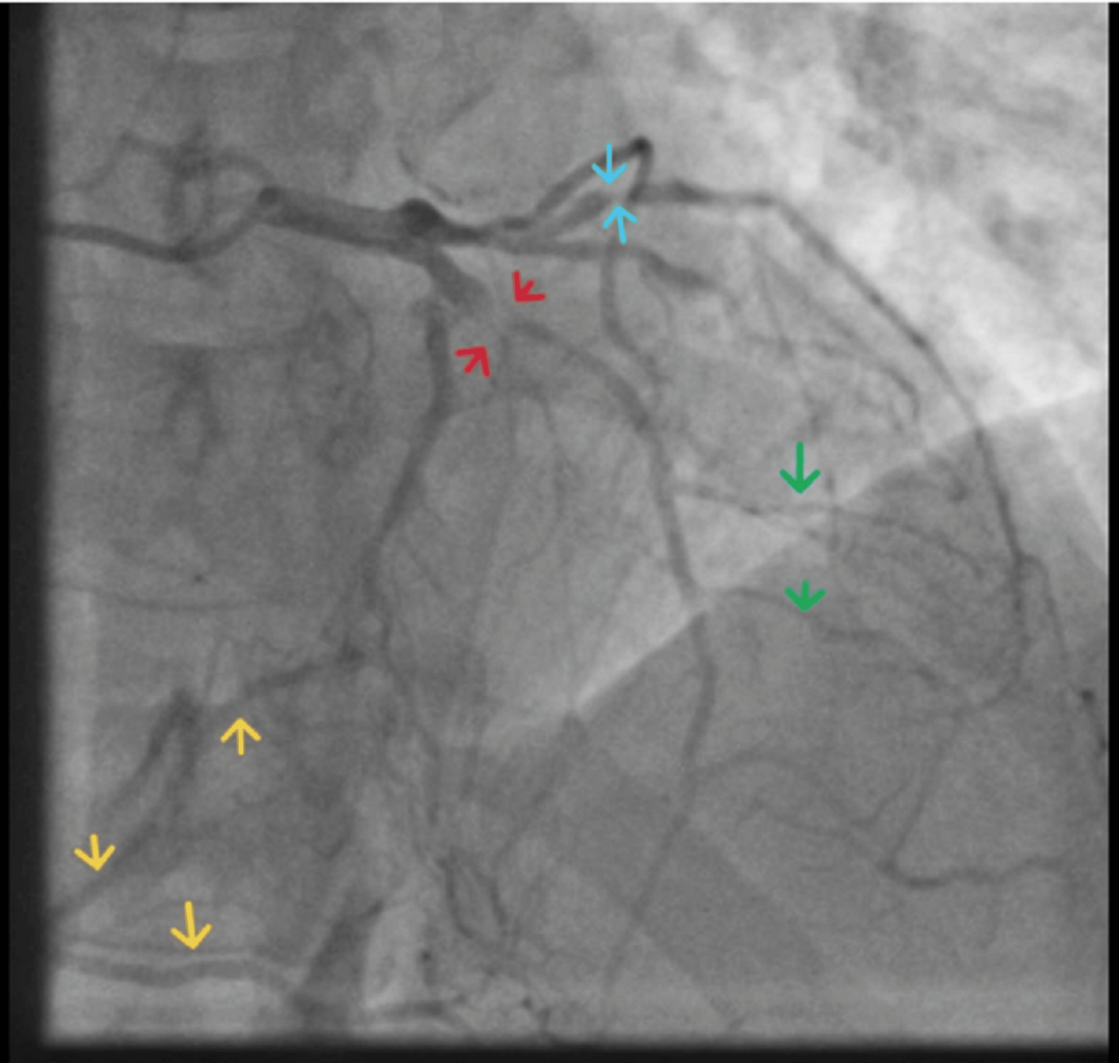
Coronary angiogram Coronary angiogram slice demonstrating collateral backfilling from the left coronary circulation to the occluded RCA (yellow arrows). Green arrows indicate diagonal branches of the LAD artery, sky blue arrows denote severe disease in the OM branch, and red arrows highlight complete total occlusion with retrograde filling of the LAD. LAD, left anterior descending; RCA, right coronary artery; OM, obtuse marginal

He was transferred to the catheterization laboratory (Cath lab) for a diagnostic invasive coronary angiogram. The procedure was performed without sedation, using standard iohexol (iodine-containing) contrast. The total contrast volume used was approximately 120 mL with controlled injection. The iodine concentration was 350 mg I/mL, with low viscosity compared to plasma at 37°C. Severe multivessel CAD was identified, as illustrated in Figure 2 and Figure 3.

The invasive diagnostic coronary angiogram demonstrated proximal occlusion of the RCA with significant collateral backfilling from the left coronary circulation. There was also a chronic total occlusion of the proximal LAD artery, along with mild disease affecting the circumflex artery. LV angiography confirmed moderately preserved systolic function, with an estimated ejection fraction (EF) of 45-50%, and acceptable diastolic function. LVEDP was elevated at 26 mmHg. No significant transaortic valve gradient was observed. Invasive blood pressure was 114/68 mmHg, with no hypotensive episodes recorded during the procedure. Arterial pressure tracings were good, and no major damping was noted. The procedure was otherwise uneventful aside from the coronary findings described.

Shortly after the procedure, the patient developed a productive cough with frothy, blood-stained sputum and acute shortness of breath. He denied chest pain. His respiratory rate was 28 breaths per minute, and oxygen saturation decreased to 90% on room air, improving to 94% on 4 L/min supplemental oxygen. Cardiovascular examination revealed normal, regular heart sounds, a heart rate of 94 bpm, blood pressure of 153/86 mmHg, and mild jugular venous pressure (JVP) elevation. Pulmonary auscultation revealed mild bilateral crepitations; no wheeze was heard. No rash was observed. The temperature was 37°C, and he was fully alert.

Laboratory investigations showed a mildly elevated CRP of 9 mg/L, while CBC, serum electrolytes, coagulation profile, and thyroid function tests remained within normal limits. Creatinine was only slightly elevated, and the eosinophil count was normal. Table 1 presents laboratory results immediately post-angiography compared to one week later.

**Table 1:** Blood tests on admission compared with repeat tests at follow-up aPTT, activated partial thromboplastin time; HCT, hematocrit; INR, international normalized ratio; MCH, mean corpuscular hemoglobin; MCV, mean corpuscular volume; RDW, red cell distribution width; PT, prothrombin time

Test	Initial result	Repeat result (week later)	Unit	Normal range
CRP	9	2	mg/L	<5
CBC				
Hemoglobin	148	149	g/L	130-180
White cell count	9.8	7.3	10⁹/L	4.0-11.0
Platelet count	196	207	10⁹/L	150-400
HCT	0.42	0.43	L/L	0.40-0.52
RCC	4.44	4.48	10¹²/L	4.5-6.5
RDW	14.3	13.7	%	11.0-14.8
MCV	94.3	95	fL	80-100
MCH	33.2	32.7	pg	27.0-32.0
Differential count				
Neutrophil count	7.14	4.08	10⁹/L	1.7-7.5
Lymphocyte count	1.69	2.34	10⁹/L	1.0-4.5
Monocyte count	0.83	0.59	10⁹/L	0.2-0.8
Eosinophils	0.09	0.23	10⁹/L	0.0-0.4
Basophils	0.05	0.05	10⁹/L	0.0-0.1
Full clotting screen				
PT	11.4	11.8	seconds	10.3-13.3
INR	1	1	INR	0.8-1.2
Derived fibrinogen	6.23	5.36	g/L	2.00-5.30
aPTT	28.6	31.7	seconds	25.7-35.3
aPTT ratio	0.96	1.06	1/1	0.8-1.2
Urea and electrolytes				
Sodium	138	141	mmol/L	133-146
Potassium	4	4.3	mmol/L	3.5-5.3
Urea	6.9	6.4	mmol/L	2.5-7.8
Creatinine	128	127	µmol/L	59-117

The primary differential diagnoses were APE secondary to ischemic heart disease and contrast-induced pulmonary edema. The management plan included admission for observation, a chest radiograph, and a focused echocardiogram. Other possible differentials were considered, including acute respiratory distress syndrome, which was less likely given that the patient had no recent infection or systemic inflammatory trigger, no history of aspiration or trauma, and stable blood pressure. Although the CXR showed bilateral infiltrates, echocardiography revealed impaired LV systolic function, with an EF of approximately 40%, making cardiogenic APE the more likely diagnosis.

Although anaphylaxis or an anaphylactoid reaction can cause acute respiratory distress through increased capillary permeability and pulmonary capillary leakage, there were no signs of urticaria, angioedema, or hypotension in this patient. Additionally, the elevated LVEDP suggested increased left-sided filling pressures. In contrast, anaphylaxis or sepsis is more commonly associated with low to normal LVEDP due to systemic vasodilation and reduced preload and afterload. Furthermore, the patient demonstrated an excellent clinical response to GTN and furosemide, supporting a cardiogenic mechanism.

PE may also mimic APE by presenting with sudden dyspnea and hypoxia and, rarely, may be associated with pulmonary edema or infarction. However, this patient did not report pleuritic chest pain. Transthoracic echocardiography showed no features of acute right ventricular strain and was not suggestive of acute PE. Additionally, he had a low Wells score and no prior history of thromboembolic disease.

Given the high likelihood of cardiogenic APE, intravenous nitrates were initiated to reduce preload and afterload, and the patient was subsequently switched to intravenous furosemide 80 mg daily the following day. He continued his regular medications, including aspirin 75 mg daily and atorvastatin 80 mg daily, which had been prescribed in the cardiology outpatient clinic. Although objective hemodynamic and imaging data were limited, the rapid clinical improvement following nitrate therapy was consistent with a cardiogenic process.

**Figure 4:**
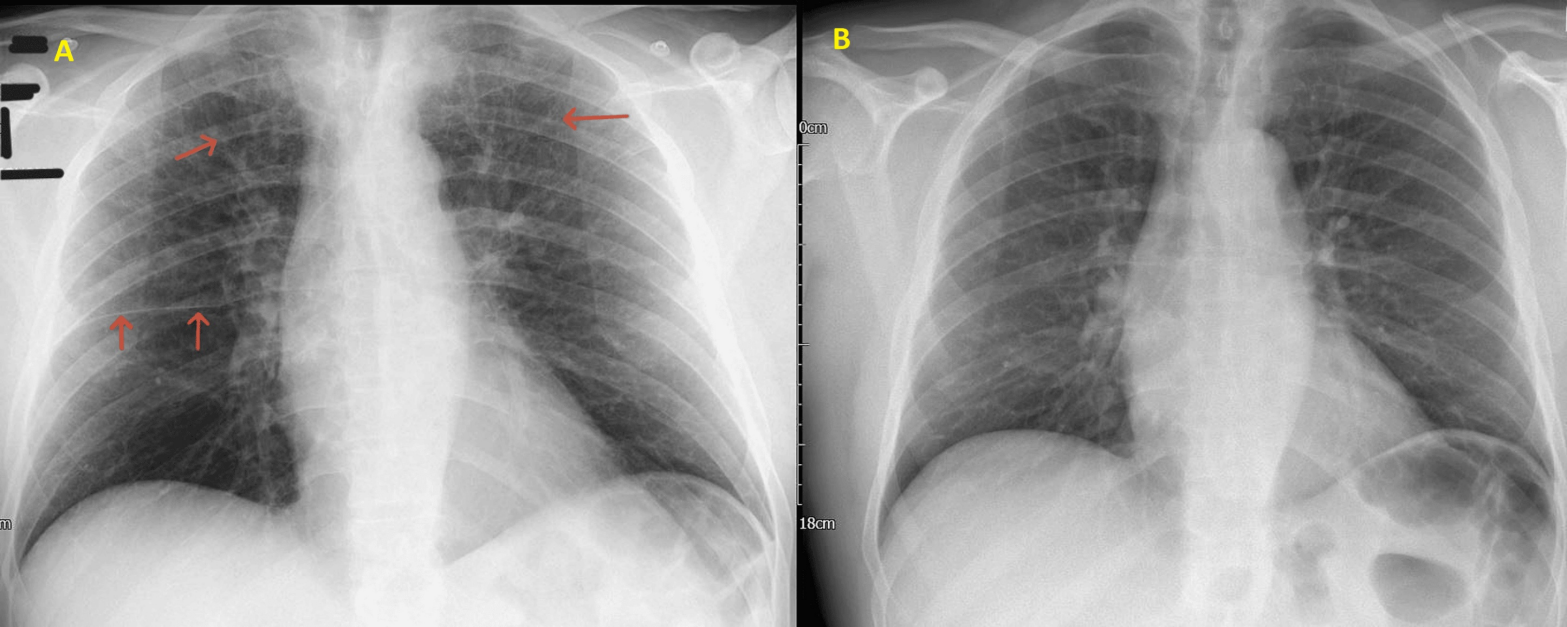
Post-angiogram CXR (A) Post-angiogram CXR showing accentuated interstitial markings in both upper zones (orange arrows at the apices) and fluid within the right horizontal fissure (Kerley B lines, red arrows). (B) Comparison with the patient’s normal CXR two months earlier, showing no abnormal findings. CXR, chest X-ray

**Figure 5:**
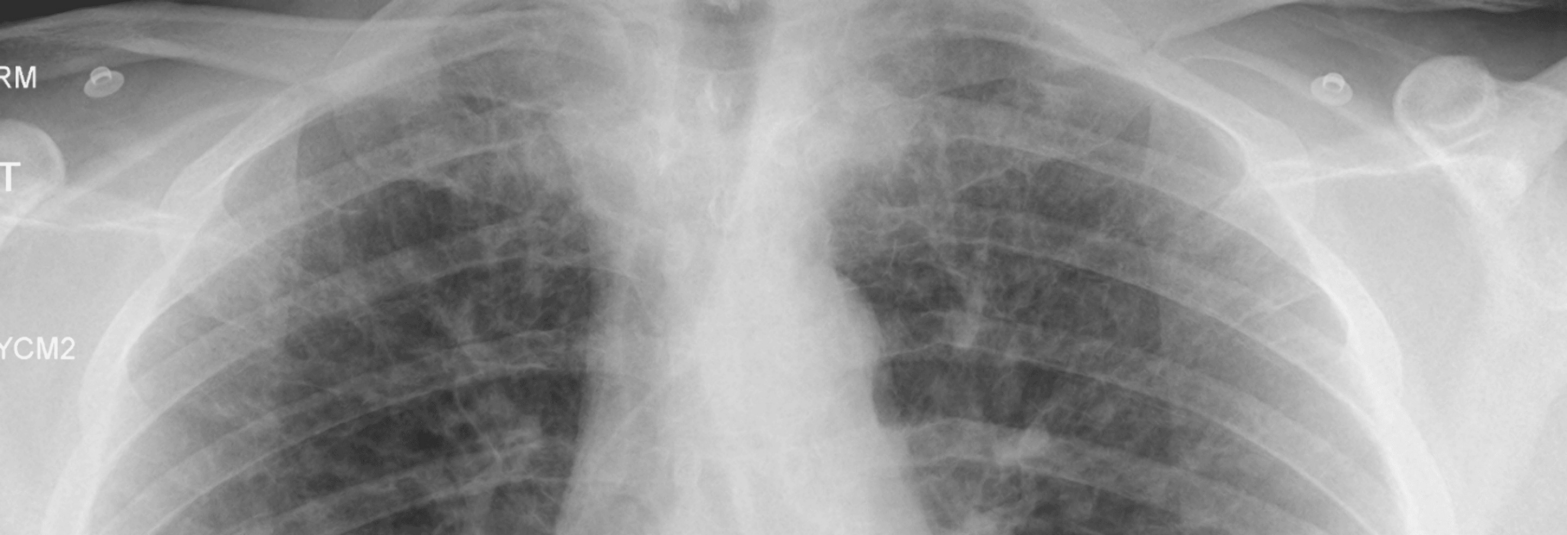
Post-angiogram CXR showing the apical zones only Accentuated lung markings are seen in the upper zones. CXR, chest X-ray

**Figure 6:**
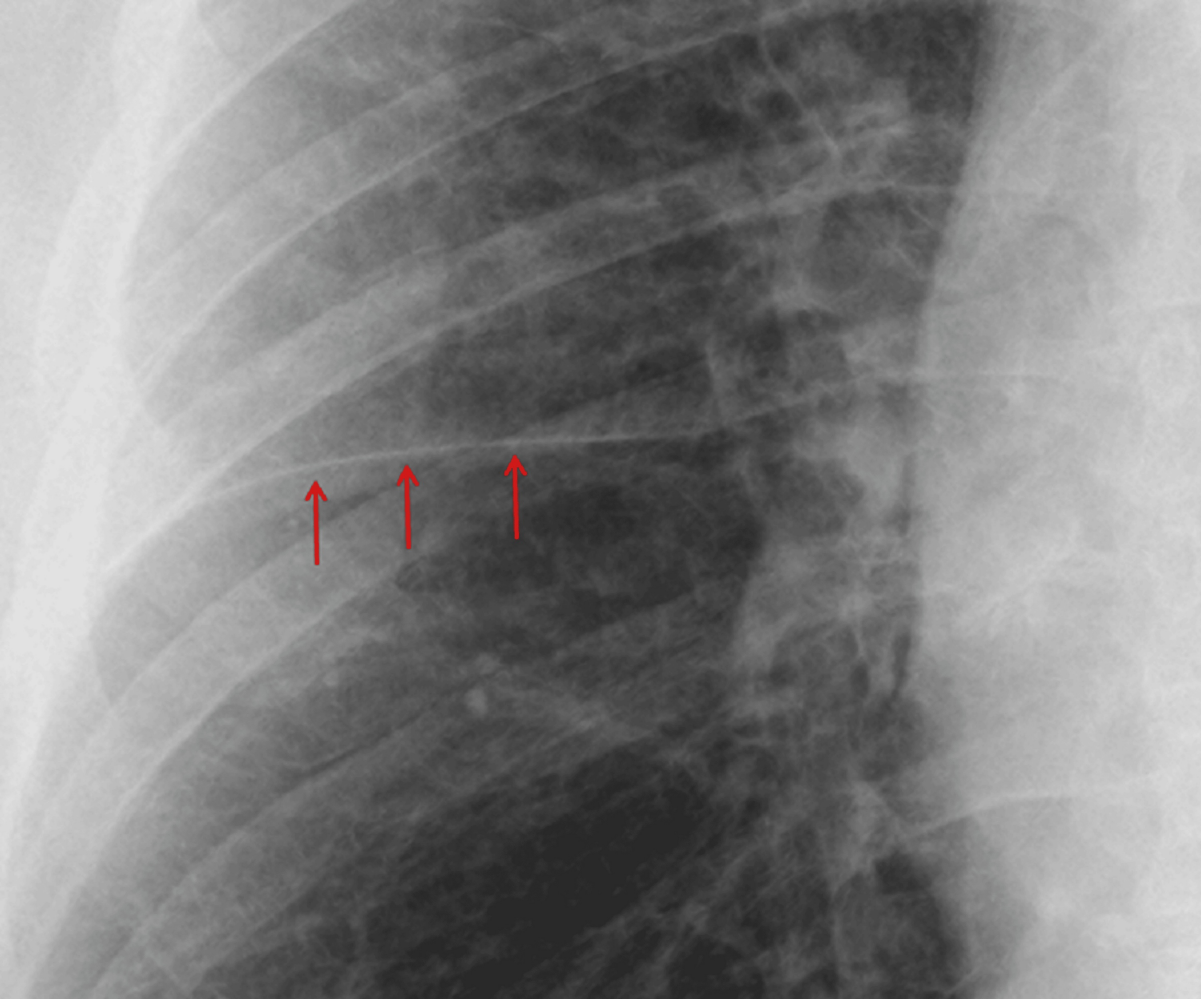
Post-angiogram CXR showing Kerley B lines in the lower zone Accentuation of the right horizontal fissure (red arrows) indicates the presence of interstitial fluid, consistent with Kerley B lines. CXR, chest X-ray

The initial chest radiograph, obtained on the day of admission, supported these differential diagnoses (Figure 4). It demonstrated accentuated interstitial markings in both upper zones, suggestive of pulmonary interstitial edema, and fluid within the right horizontal fissure (Kerley B lines) (Figure 5, Figure 6).

The echocardiogram performed following the diagnostic coronary angiography demonstrated mild impairment of LV systolic function, most prominent in the inferior and posterior walls. The visually estimated EF was approximately 40%, representing a mild reduction from the initial echocardiogram performed a few weeks earlier. The remainder of the study was unremarkable, apart from the presence of mild mitral regurgitation.

The patient demonstrated excellent clinical recovery, with resolution of cardiorespiratory symptoms. Within 48 hours, he had returned to baseline. Vital signs were as follows: heart rate 68 bpm, normal heart sounds, normal JVP; respiratory rate 16 breaths per minute; oxygen saturation 98% on room air (FiO₂ 0.21). Pulmonary auscultation revealed normal vesicular breath sounds, and the patient was functionally in New York Heart Association (NYHA) class II. These findings further supported a diagnosis of acute cardiogenic pulmonary edema, helping to exclude contrast-induced reactions or noncardiogenic APE.

**Figure 7:**
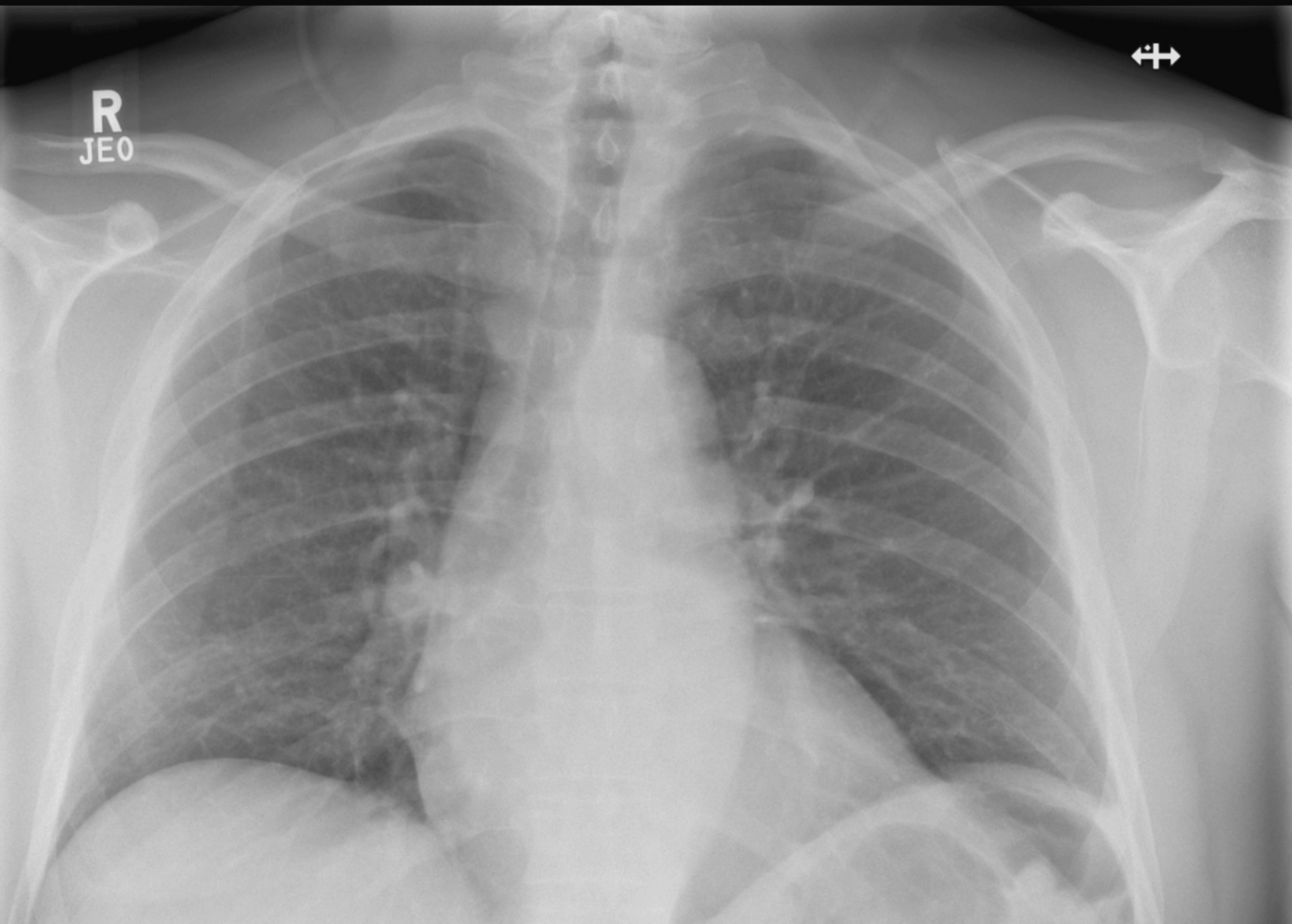
Follow-up chest radiograph obtained three days after the APE episode, demonstrating interval resolution of pulmonary congestion APE, acute pulmonary edema

Follow-up chest radiography three days later showed clear lung markings with resolution of Kerley B lines (Figure 7).

The patient was discussed in a multidisciplinary meeting involving the interventional cardiology and cardiothoracic surgery teams. Coronary artery bypass grafting (CABG) at the tertiary care center was deemed appropriate based on the identification of three-vessel CAD on coronary angiography and the presence of suitable distal bypass targets. The SYNTAX II score was 24, indicating intermediate risk; however, CABG was unanimously considered to offer better four-year mortality outcomes compared with percutaneous coronary intervention/angioplasty.

The operation was performed on-pump and involved a pedicled left internal mammary artery graft to the LAD artery and reversed saphenous vein grafts to the posterior descending artery and obtuse marginal branch, all supplying moderately diseased vessels. Postoperatively, the patient remained in sinus rhythm with mildly reduced LV function and moderate mitral regurgitation. He was transferred to the high-dependency unit for monitoring, remained stable, and was subsequently stepped down to the ward. No ventilatory or circulatory support was required, and vital signs remained stable. The surgical wound was clean and intact. The cardiothoracic team discharged him on the fifth postoperative day, with a follow-up appointment scheduled in the outpatient clinic three months later.

Additionally, the cardiology team arranged post-CABG follow-up to monitor recovery and ongoing cardiac care. His recovery was satisfactory, with no symptoms suggestive of decompensated heart failure. The patient was reviewed in the post-discharge Heart Failure Clinic one month after CABG. At review, he was recovering well, with stable vital signs (blood pressure 134/86 mmHg, heart rate 70 bpm, weight 85.9 kg) and no clinical signs of fluid overload or respiratory compromise. Repeat echocardiography demonstrated further reduction in LV systolic function, with an LVEF of 35%, and electrocardiography confirmed sinus rhythm.

His furosemide dose was reduced to 20 mg once daily. Ramipril 2.5 mg once daily and bisoprolol 2.5 mg once daily were introduced. He continued daily therapy with aspirin and a statin. A mineralocorticoid receptor antagonist (MRA) was planned to commence in one week, depending on his response to initial medications and electrolytes. Blood tests were scheduled to be repeated in one to two weeks, and follow-up with the Community Heart Failure Team was planned. He was scheduled for a general cardiology clinic review in three months to assess LV function recovery and to consider the introduction of additional guideline-directed medical therapy (GDMT) for heart failure, including sacubitril/valsartan and an SGLT2 inhibitor.

The team emphasized the importance of individualizing the four pillars of therapy for heart failure with reduced EF (HFrEF) based on patient tolerance and clinical response. A phased approach to optimizing medications was implemented. The four pillars of HFrEF GDMT include a renin-angiotensin-aldosterone system inhibitor, a beta-blocker, an MRA, and an SGLT2 inhibitor.

Outpatient cardiac rehabilitation was arranged post-CABG, and the patient attended with good compliance. Rehabilitation spanned six weeks, during which he improved to NYHA class I by the end of the program.

## Discussion

APE is an uncommon but potentially life-threatening complication following diagnostic coronary angiography. Reported data vary, but the incidence of cardiogenic APE related to coronary angiography is low (<2%), while contrast-mediated noncardiogenic APE has been reported as rare as 0.001-0.008% [4]. This case of a 60-year-old man with known CAD developing APE shortly after a diagnostic angiogram underscores the importance of vigilance for this uncommon but critical adverse event. While the procedure is generally considered safe, cardiopulmonary events such as arrhythmias, myocardial infarction, contrast-induced nephropathy, and, rarely, APE have been described [5,6]. Pulmonary edema post-angiography may arise from multifactorial and overlapping mechanisms, including transient ischemia-induced LV dysfunction, catheter-induced coronary spasm, volume overload, or contrast-mediated endothelial injury, increasing capillary permeability.

Multiple, not mutually exclusive, mechanisms may explain pulmonary edema after coronary angiography: (1) acute cardiogenic pulmonary edema due to transient myocardial ischemia, catheter-induced coronary spasm, or worsening LV systolic/diastolic dysfunction with a rise in LV filling pressures; (2) contrast-mediated endothelial injury and increased capillary permeability producing a noncardiogenic capillary leak; and (3) volume or contrast overload precipitating decompensation in patients with limited cardiac reserve. Distinguishing cardiogenic from noncardiogenic mechanisms has important prognostic and therapeutic implications. As observed in our case, cardiogenic edema typically responds to preload and afterload reduction and inotropes as needed, whereas noncardiogenic pulmonary edema (NCPE) may require more intensive respiratory support and attention to inflammatory or vascular permeability mechanisms [7,8].

The patient responded promptly to intravenous nitrate infusion followed by diuresis, without requiring mechanical or circulatory support. This clinical course makes an anaphylactoid reaction or NCPE unlikely. A further diagnostic clue was the elevated LVEDP of 26 mmHg, indicating raised left-sided diastolic pressures, which is more consistent with forward LV failure (LV systolic dysfunction) than with noncardiogenic causes of pulmonary edema, such as anaphylaxis or sepsis, in which LVEDP is typically low to normal due to reduced preload and afterload. Potential risk modifiers in his case include the need for more optimal preprocedural diuresis, as well as minimizing both the contrast volume and procedure duration. The latter could be achieved by acquiring fewer but more targeted and diagnostically relevant images.

Clinical observations by Kang and Nah demonstrated that nonionic, low-osmolar radiological contrast media, such as that used in this case, can directly alter pulmonary vascular permeability, promoting interstitial and alveolar fluid accumulation independent of overt LV dysfunction [9]. This provides mechanistic support for noncardiogenic contrast-mediated pulmonary edema via endothelial injury and capillary leak pathways. More recently, Rimoldi et al. described a case of flash pulmonary edema occurring in the cardiac catheterization laboratory, attributed to abrupt rises in LV filling pressures and acute hemodynamic stress during angiography [10]. Their report underscores the importance of procedural vigilance, early recognition, and immediate hemodynamic stabilization in susceptible patients.

Predisposing factors for APE include preexisting CAD, impaired LV function, renal dysfunction, and procedural variables such as high contrast volume or hemodynamic stress. In this case, although the patient was euvolemic on examination prior to left heart catheterization, severe multivessel coronary disease was only identified on angiography. The most plausible mechanism for his APE is a transient ischemic effect induced by contrast injection in an already under-perfused myocardium, precipitating acute LV impairment and fluid overload, particularly in the recumbent position due to a sudden increase in preload.

Although he had a preexisting mildly impaired EF (estimated LVEF 45%), contrast-mediated endothelial effects and volume/contrast burden cannot be excluded as contributing or amplifying factors. The favorable and rapid response to IV nitrates and continued diuresis supports a dominant cardiogenic mechanism in this instance, although mixed mechanisms may coexist. The contrast used was iohexol, a low-osmolar, nonionic iodinated agent with low viscosity, administered via controlled infusion. The iodine concentration was 350 mg I/mL, slightly hypertonic to plasma but generally considered safe and well-tolerated. Importantly, the patient was not under any sedation during the procedure.

From a preventative standpoint, careful preprocedural assessment, including evaluation of LV function, optimization of volume status, and minimization of contrast volume, is essential. Additionally, procedural sedation and blood pressure control may mitigate sympathetic surges contributing to acute decompensation. This case highlights the need for heightened clinical suspicion for pulmonary edema in patients undergoing invasive cardiac procedures, particularly those with significant CAD and compromised ventricular function. Although rarely reported, APE is a potential complication to consider during patient consenting and procedural preparation.

## Conclusions

Acute (cardiogenic) pulmonary edema following diagnostic coronary angiography, although uncommon, represents a serious clinical condition that requires prompt recognition and intervention. Awareness of predisposing factors, such as severe coronary disease and LV impairment, is important, as these may lead to this complication even in stable, euvolemic patients, as demonstrated in our case. Understanding the underlying pathophysiological mechanisms and implementing timely management strategies are essential to optimize patient outcomes.

Further research is warranted to clarify the precise mechanisms, particularly in cases of noncardiogenic APE mediated by contrast, and to develop effective preventive measures. Anticipating and implementing preparatory measures prior to the procedure is essential. From a medicolegal and patient safety standpoint, identifying patients at risk requires appropriate education and thorough informed consent. Patients should be made aware of the risks, especially if they have a history of hypersensitivity to iodine-containing contrast agents and/or heart failure symptoms, regardless of their NYHA class. We aim to provide further follow-up updates at six and 12 months postoperatively.
